# Membrane Filtration of Nanoscale Biomaterials: Model System and Membrane Performance Evaluation for AAV2 Viral Vector Clarification and Recovery

**DOI:** 10.3390/nano15040310

**Published:** 2025-02-18

**Authors:** Mara Leach, Kearstin Edmonds, Emily Ingram, Rebecca Dutch, Ranil Wickramasinghe, Malgorzata Chwatko, Dibakar Bhattacharyya

**Affiliations:** 1 Department of Chemical and Materials Engineering, University of Kentucky, Lexington, KY 40506, USA; mle271@uky.edu (M.L.);; 2 College of Medicine, University of Kentucky, Lexington, KY 40506, USA; kearstin.edmonds@uky.edu (K.E.);; 3 Department of Chemical Engineering, University of Arkansas, Fayetteville, AR 72701, USA; swickram@uark.edu

**Keywords:** AAV2, silica nanoparticles, membrane filtration, fouling models, downstream bioprocessing, gene therapy

## Abstract

The growing demand for viral vectors as nanoscale therapeutic agents in gene therapy necessitates efficient and scalable purification methods. This study examined the role of nanoscale biomaterials in optimizing viral vector clarification through a model system mimicking real AAV2 crude harvest material. Using lysed HEK293 cells and silica nanoparticles (20 nm) as surrogates for AAV2 crude harvest, we evaluated primary (depth filters) and secondary (membrane-based) filtration processes under different process parameters and solution conditions. These filtration systems were then assessed for their ability to recover nanoscale viral vectors while reducing DNA (without the need for endonuclease treatment), protein, and turbidity. Primary clarification demonstrated that high flux rates (600 LMH) reduced the depth filter’s ability to leverage adsorptive and electrostatic interactions, resulting in a lower DNA removal. Conversely, lower flux rates (150 LMH) enabled >90% DNA reduction by maintaining these interactions. Solution conductivity significantly influenced performance, with high conductivity screening electrostatic interactions, and the model system closely matching real system outcomes under these conditions. Secondary clarification highlighted material-dependent trade-offs. The PES membranes achieved exceptional AAV2 recovery rates exceeding 90%, while RC membranes excelled in DNA reduction (>80%) due to their respective surface charge and hydrophilic properties. The integration of the primary clarification step dramatically improved PES membrane performance, increasing the final flux from ~60 LMH to ~600 LMH. Fouling analysis revealed that real AAV2 systems experienced more severe and complex fouling compared to the model system, transitioning from intermediate blocking to irreversible cake layer formation, which was exacerbated by nanoscale impurities (~10–600 nm). This work bridges nanomaterial science and biomanufacturing, advancing scalable viral vector purification for gene therapy.

## 1. Introduction

The clinical success of gene therapy relies on producing high-quality viral vectors such as adeno-associated viruses (AAV), which are nanoscale particles (~20–25 nm) used to deliver therapeutic genes. However, the purification of viral vectors from cell culture harvests remains a significant challenge due to coexisting impurities, such as host cell proteins, DNA, and cell debris. Traditional clarification techniques often face trade-offs between product recovery and impurity removal, necessitating the development of scalable and efficient purification strategies.

### 1.1. AAV Basics

AAV was first discovered in the 1970s by Atchison et al. and is a small non-enveloped single-stranded DNA (ssDNA) virus categorized as a parvovirus [1,2]. AAV has a diameter of 20–25 nm and has become the most used vector for gene therapies due to its safety, efficacy, and ability to target specific tissues. To date, ten viral vector-based therapies have been approved by the FDA, eight of which use AAVs (i.e., Glybera, Luxturna, Zolgensma, Upstaza, Roctavian, Hemgenix, Elevidys, and Elaparvovec) [1,3]. There are 13 human stereotypes of AAVs that differ based on capsid proteins and whether they are expressed intracellularly or secreted during upstream processes. For example, AAV9 and lentivirus, another common viral vector, are primarily secreted into the cell culture supernatants and can be directly clarified without cell lysis [1,2]. This imparts the ability to incorporate a perfusion clarification system, like that which Tona et al. developed for lentivirus manufacturing [4]. However, most other AAVs, as well as adenoviruses, are expressed intracellularly, and the release of these particles requires cell lysis conducted using chemical lysis agents such as Tween 20 or Triton X-100 surfactants, freeze–thaw cycles, or sonication [1,2]. While this step is critical for obtaining high yields, it also introduces substantial impurities into the crude harvest, including cellular debris, DNA, and host cell proteins. These impurities pose significant challenges for clarification processes, leading to increased operational complexity, higher costs for downstream DNA removal, and a reduced process efficiency. Effective clarification methods are therefore essential to improve recovery and reduce the costs associated with AAV production.

### 1.2. Current Clarification Techniques and Limitations

Centrifugation is one of the conventional methods used for viral vector clarification. However, this approach is often cost-prohibitive and inefficient for large-scale production. The process suffers from limited throughput, labor-intensive workflows, and challenges in scaling it up for industrial applications [5]. Recent advances in centrifugation have been made by Wada et al., who investigated a two-step cesium chloride (CsCl) density gradient ultracentrifugation with a zonal rotor for large-scale (1000 L) AAV purification [6]. Despite this, centrifugation often leads to incomplete separation, leaving behind residual impurities that can affect downstream processes and result in the significant loss of valuable viral vectors [7,8,9,10,11,12].

Depth filters are employed for the clarification of viral vectors, particularly at a large scale, due to their ability to handle high turbidity and large volumes efficiently. For instance, depth filters have demonstrated success in the clarification of lentivirus harvest, achieving high recovery rates and significant reductions in turbidity [13]. Similarly, A. Venkiteshwaran et al. evaluated various commercial depth filters for viral clearance, highlighting their adsorptive properties for host cell proteins (HCP) and DNA, which are major impurities in viral vector processes [14]. This work has been further corroborated by studies from O. Khanal et al., Chu et al., and N. Borochov, who investigated the depth filter performance under different process conditions and impurities [14,15,16].

Expanding on this, Meierrieks et al. explored the use of filter aids such as diatomaceous earth for AAV clarification via depth filtration and compared their performance to centrifugation [2]. They observed that filter aids significantly improved filter capacity and decreased the operational time, highlighting their potential utility for viral vector clarification.

Despite these advances, the clarification step for AAV production remains a major cost driver, contributing up to 50 times the cost of any other downstream process [17]. For a 250 L bioreactor, this translates to an estimated cost of 49 million USD. A substantial portion of this expense is attributed to the use of endonuclease treatments, which are required to degrade host cell and plasmid DNA post-cell lysis [17,18,19]. Endonuclease treatments alone can account for 51% of the total cost [17]. However, there is limited research exploring clarification techniques that minimize or eliminate the need for endonuclease, which presents a critical opportunity for cost reduction. Thakur et al. demonstrated an endonuclease-free approach via chromatography that showed cost savings of 100,000 USD per 500 L batch for AAV8/9 clarification [20]. Similar work has not been demonstrated via other separation processes or AAV2.

While substantial progress has been made in understanding the adsorptive properties of depth filters for proteins and DNA, these insights have largely been limited to systems involving individual components’ or monoclonal antibodies’ (mABs) production. For example, Chu et al. evaluated the binding characteristics of model proteins with varying charge and hydrophobicity across two depth filters with differing properties [16]. However, in the complex environment of viral vector clarification—where competing nanoscale biomaterials such as viral particles, DNA, and proteins coexist—adsorptive and size-exclusion mechanisms have not been systematically studied.

Flow rate is another key factor influencing depth filter performance. M. Parau et al. investigated the effect of varying flow rates on commercial depth filters during mAb production and observed significant impacts on both filter capacity and DNA breakthrough [21]. However, similar studies for viral vector clarification remain absent. Given that viral vectors like AAVs carry a net negative charge, their interactions with depth filter media may yield different results compared to proteins or DNA alone, particularly under varying flow rates.

Furthermore, the underlying mechanisms of impurity removal—whether through size exclusion or adsorptive behavior—have not been explored in the context of viral vector clarification. Such insights are essential for optimizing depth filter selection and operating conditions.

To date, there is also a significant knowledge gap in the systematic evaluation of depth filter properties for AAV2 clarification. Key properties such as pore size distribution, ionic capacity (charge-based behavior), and material composition have not been thoroughly investigated in relation to throughput, impurity removal, and recovery rates. While studies such as A. Venkiteshwaran et al. have analyzed the ionic capacity of depth filters and the solution conductivity effects on virus retention for parvovirus clearance, the primary objective in these studies was virus removal, rather than maximizing throughput and recovery—critical metrics for viral vector production processes [14].

Tangential flow filtration (TFF) is a widely utilized membrane filtration technique in bioprocessing, known for its scalability and efficiency. While extensively applied for protein purification, TFF has also been investigated for the concentration and sterile filtration of viral vectors using ultrafiltration membranes. For example, Doria et al. demonstrated the efficacy of TFF for AAV concentration, achieving a 60 h reduction in the processing time compared to CsCl-gradient ultracentrifugation [22]. Miyaoka et al. evaluated TFF for AAV concentration post-clarification and reported a high protein and DNA clearance, with the addition of surfactants further enhancing protein removal [23]. Reviews by Besnard et al. and Kilgore et al. have extensively highlighted the use of TFF and normal flow filtration (NFF) for both the clarification and downstream purification of viral vectors in vaccine production and gene therapy [1,24]. These works emphasize the versatility and efficiency of membranes in achieving high product quality.

Fouling is a well-documented challenge in membrane systems, especially when processing crude harvests rich in impurities. The accumulation of fouling layers can significantly reduce the membrane efficiency and throughput. To mitigate fouling, TFF systems often employ an increased crossflow velocity or flow rate to reduce the accumulation of foulants on the membrane surface. However, in the context of delicate viral vectors, high shear rates associated with increased flow velocities pose a significant risk of damaging the viral particles, compromising recovery and product integrity [9,25,26,27,28,29,30,31]. Furthermore, while cleaning procedures and membrane reuse are common in other applications, single-use membranes are typically employed for viral vector purification due to sterility and cross-contamination concerns. This makes understanding fouling dynamics in this context even more critical. These limitations underscore the necessity of implementing a primary clarification step, such as depth filtration, to alleviate the fouling burden and improve the membrane performance without exposing the viral vectors to harmful shear conditions, and to ensure sterility.

### 1.3. Nanoscale Synthetic Model for Filtration Evaluation

Synthetic models that mimic the properties of viral vector systems have gained attention due to their ability to simplify the evaluation of filtration techniques. For instance, nanoparticles of defined size ranges can serve as surrogates for AAV, enabling controlled and reproducible studies. For example, nanoparticles have been used to model lentivirus for purification and for evaluating the retention and fouling behavior of membranes [32,33]. Furthermore, in our previous work, we evaluated the impact of membrane morphologies on nanoparticle and protein purification, and the implications for AAV2 purification [34]. In this context, we selected silica nanoparticles as the model system for our study due to their well-defined size distribution, chemical stability, and widespread use in virus filtration research [32,33,35,36,37,38]. While unmodified synthetic particles like silica nanoparticles are commonly employed in the literature to simulate viruses, direct comparisons with real AAVs have not been extensively investigated. This work aims to bridge that gap by assessing the suitability of synthetic nanoparticles as models for AAV2 filtration and identifying the similarities and differences in filtration behavior under comparable conditions, providing highly valuable insights into the validity of using synthetic nanoparticles as surrogate particles in viral filtration studies.

### 1.4. Aims/Objectives

To address the critical gaps in the literature, this study aims to conduct the following:

1. Evaluate the performance of depth filters as a primary clarification under varying flow rates, conductivity/salinity conditions, and material properties for AAV2 clarification, as well as investigate the mechanisms of impurity removal without the addition of any endonuclease.
2. Analyze the effectiveness of secondary membrane filtration (PES and RC membranes) following primary clarification, focusing on recovery, impurity removal, and fouling mechanisms for single-use applications.
3. Compare a synthetic nanoparticle-based model system to a real AAV2 crude harvest to assess its suitability for evaluating filtration performance data.

By addressing these objectives, this study provides a comprehensive investigation into the clarification of AAV2 using a combined depth filtration and membrane filtration approach, offering new insights for optimizing viral vector production processes.

## 2. Materials and Methods

### 2.1. Materials

Cell culture reagents (Dulbecco’s Modified Eagle Medium (DMEM), phosphate-buffered saline (PBS)), a protein (Bradford) assay kit, and a DNA assay kit were obtained from Thermo Fisher Scientific (Carlsbad, CA, USA). The depth filters CE50 (cellulose fibers) (MilliporeSigma, MCE5023CL3) and X0SP (synthetic polyacrylic fibers with silica filter aid) (MilliporeSigma, MD0SP23CL3) were from MilliporeSigma (Saint Louis, MO, USA). The PES flat sheet membranes were obtained from Sterlitech (Charlotte, NC, USA). The RC membrane flat sheet membranes were from Sartorius (Bohemia, NY, USA). The AAV2 crude harvest, used for comparison with the model system, was purchased from The University of North Carolina (UNC) Vector Core (Chapel Hill, NC, USA). The 20 nm fluorescently labeled (via fluorescein isothiocyanate (FITC)) silica nanoparticles with an Ex/Em of 495 nm/520 nm which were used as a model AAV2 (mAAV2) were from Superior Silica (Chandler, AZ, USA) and were supplied in deionized water. The sodium chloride and sodium phosphate were purchased from VWR.

### 2.2. Cultivation of HEK293 Cells

HEK293T cells (ATCC, Gaithersburg, MD, USA, CRL-3216) were grown on T175 flasks (Greiner, Monroe, NC, USA, 660175) in DMEM (Corning, Harrodsburg, KY, USA, 10-013-CV). T175 flasks generate approximately 1.75 × 10^7^ cells per flask. To mimic a volume produced by a suspension culture, 23 flasks (per experimental replicate) were grown for a total cell count of ~4 × 10^8^. The process was completed in triplicate. The cells were lifted with trypsin (Life Technologies, Frederick, MD, USA, 15400-054) for 5 min, resuspended in DMEM, and centrifuged at 3200× *g* for 5 min. The cell pellets were resuspended in PBS (Life Technologies, Fredrick, MD, USA, 20012050) and centrifuged again using the same parameters. The cell pellets were then lysed at 65 °C for 15–30 min, depending on the pellet size, before being frozen at −20 °C prior to the experiments.

### 2.3. Model Crude Harvest

The model crude harvest was prepared using fluorescent silica nanoparticles, to simulate AAV2 viral vectors offering similar properties, as outlined in Table 1, and lysed HEK293T cells. mAAV2 were prepared at a concentration of 5 × 10^14^ particles/mL, as is expected for real AAV2 crude harvests. The lysed HEK293T cell pellet was mixed with the fluorescent silica nanoparticle solution in DMEM to create the model crude harvest. The mixture was stirred for 12 h. at 4 °C to ensure a homogeneous distribution of nanoparticles and cellular debris. This model crude harvest, designed to simulate the complexity of AAV2 crude harvest, was then used for the subsequent filtration and characterization experiments.

### 2.4. Depth Filtration for Primary Clarification

For primary clarification, two depth filters were evaluated: Filter A, a high-capacity filter with a nominal pore size of 1 to 0.6 µm composed of cellulose fibers, and Filter B, a filter with a nominal pore size of 9 to 0.6 µm and composed of polyacrylic fibers (16.5 ± 1.4 µm in size for largest layer [21]). The filters were chosen for their pore size distribution, material composition, and applicability for use as a primary clarification material, representing commonly used materials used during the clarification of crude harvests [1,2,24,42]. Filtration was conducted using a laboratory-scale depth filtration equipped with pressure sensors before and after the filtration module and a peristaltic pump for controlling the flow rate. Silicone tubing was used to ensure minimal product loss. The tested systems (model crude harvest and AAV2 crude harvest (used within a biosafety cabinet)) were passed through each filter at two controlled flow rates, with continuous monitoring of the pressure drop across the filters. The flow rates chosen, corresponding to constant flux rates, were 150 L·m^−2^ h^−1^ (LMH) and 600 LMH. These represent commonly used process parameters as well as recommended rates by the manufacturer, MilliporeSigma. Depth filters were only operated when having a transmembrane pressure (TMP) of less than 5 psi. The flow rate and flux rate of each filter were measured to assess the stability of the filtration process, as shown in Figure S1.

### 2.5. Ionic Capacity Analysis

The ionic capacity of the depth filters was analyzed using a counter-ion displacement method derived from Chen et al. [14]. This method is widely utilized in the literature for assessing the ionic capacities of depth filters [14,20]. The depth filters were flushed with 250 mL of 500 mM sodium chloride to exchange all positive functional groups within the filter with chloride ions. Then, the depth filters were flushed with RICCA Type I/II deionized water while the outlet solution conductivity was monitored until it reached the limit of detection (<5 µS/cm) to ensure all free counter ions were removed from the filter. Finally, the depth filters were flushed with 100 mM sodium phosphate (pH 7) while the outlet was monitored until the conductivity reached a stable value. This ensured the complete displacement of all chloride ions from the depth filter. In our calculations, we assumed that all bound chloride ions were displaced by higher charge phosphate anions during the counter-ion displacement process. Given the lower concentration of the sodium phosphate solution, the contribution of phosphate ions to the conductivity was minimal, thereby ensuring the accuracy of our ionic capacity determinations. To ensure the accuracy and reliability of our results, we validated our findings by comparing them with existing literature on the same commercial depth filters and found consistent outcomes [14,20].

The permeate volume collected during this displacement was determined using an electronic balance. The flow rate for all steps through the depth filter was set at 10 mL/min using a peristaltic pump. The [Cl^−^] concentration of the displacement samples was determined using the conductivity-based concentration curve shown in Figure S11. The depth filter’s ionic capacity was then determined using the equation below:

$$Ionic\;Capacity=\frac{\left[{Cl}^{-}\right]\times V}{A}$$

where [Cl^−^] is the chloride concentration in the permeate, V is the permeate volume collected during the displacement, and A is the depth filter area.

### 2.6. Tangential Flow Filtration (TFF) for Secondary Clarification

Secondary clarification was performed using TFF with two membrane types: PES and RC, representing commonly used membrane materials during bioprocessing. Both membranes had a pore size of ~0.2 µm and were chosen for their known performance characteristics in bioprocessing applications [24]. The experiments were conducted using a lab-scale TFF system, where the tested system, pre-treated by depth filtration (non-continuous, two-batch operation), was passed through each membrane under a constant TMP of 1 bar and a constant feed flow rate of 800 mL/min (Re = ~2300). This feed flow rate was chosen based on a preliminary analysis as well as it being a commonly used flow rate to maximize the shear rate without causing damage to viral vectors [23]. The flux through the membranes was measured by monitoring the volume of permeate collected over time using an electronic balance and Mettler Toledo BalanceLink software (237010, v1), and calculated using the equation below:

$$Flux\;(J)=\frac{Volume}{{Time\;*\;Area}_{membrane}}$$

Pure water flux, pristine scanning electron microscopy (SEM) images, and the experimental TFF setup are shown in Figures S2–S4.

### 2.7. AAV2 Crude Harvest

Transfected AAV2-producing HEK293T cells were received on dry ice and were immediately stored at −80 °C until further processing. To prepare the crude harvest, the cells were subjected to three freeze–thaw cycles to ensure efficient lysis and viral release. The cells were rapidly frozen at −80 °C and thawed at 37 °C, repeating this process a total of three times. All samples, both pre- and post-clarification, were stored at 4 °C for no more than 5 days before analysis [42]. Afterward, any remaining material was frozen at −80 °C for long-term storage to maintain sample stability before additional testing or other downstream processes.

### 2.8. Analytical Assays

#### 2.8.1. Turbidity

The turbidity of both the feed and permeate solutions was measured using a calibrated Apera Instruments (Columbus, OH, USA) TN400 portable turbidity meter as described in the Supporting Information.

#### 2.8.2. Dynamic Light Scattering

The size distribution of particles, in both the feed and permeate solutions, was determined using dynamic light scattering (DLS) analysis as described in the Supporting Information and Figure S5.

#### 2.8.3. Protein Quantification

The protein concentration of both the feed and permeate solutions was determined using a Bradford protein assay (ThermoFisher, Carlsbad, CA, USA, 23200) as described in the Supporting Information and Figure S6.

#### 2.8.4. DNA Quantification

The DNA concentration of both the feed and permeate solutions was determined using the bisBenzimide DNA Quantitation Kit (Fluorescence Assay) as described in the Supporting Information and Figure S7.

#### 2.8.5. Model AAV2 (mAAV2) Quantification

The concentration of the model AAV2 (20nm fluorescent silica nanoparticles Ex/Em 485/520 nm) was determined using a BioTeck Synergy hybrid microplate reader (Agilent Technologies, Santa Clara, CA, USA). First, a standard curve was created using the kit standard solutions of known silica nanoparticle concentrations prepared by serial dilution, obtaining an R^2^ > 0.99, as shown in Figure S8.

For the sample measurements, 100 µL of each feed and permeate sample was added to a black 96-well plate, which minimized background interference and enhanced fluorescence detection. Fluorescence measurements were conducted using a BioTeck Synergy hybrid microplate reader.

The percentage of model AAV2 recovery in the permeate was calculated using the equation below:

$$Model\;AAV2\;Recovery\left(\%\right)=\left(\frac{{Intensity}_{permeate}}{{Intensity}_{feed}}\right)\times 100$$

Each sample was measured in triplicate to ensure accuracy, and the average values were used to assess the efficiency of model AAV2 recovery in the permeate after filtration.

#### 2.8.6. AAV2 Quantification

The AAV2 concentration in both the feed and permeate was determined using an AAV2 enzyme-linked immunosorbent assay (ELISA) kit (Progen, Wayne, PA, USA, PRAAV2XP), following the manufacturer’s protocol. The assay was performed to quantify AAV2 recovery in the permeate after filtration. A standard curve was generated using the known concentrations of AAV2 capsid standards provided with the kit, as shown in Figure S9. All samples were diluted 400:1 using the ASSB buffer to fit into the readability of the plate (<2.5 × 10^9^ capsids/mL). The concentration of AAV2 in the feed and permeate samples was determined by comparing their absorbance values as per manufacturer instructions, measured on a SpectraMax iD3 plate reader (Molecular Devices, San Jose, CA, USA), to the standard curve.

The percentage of AAV2 recovery in the permeate was calculated using the equation below:

$$AAV2\;Recovery\;\left(\%\right)=\left(\frac{{AAV2\;Concentration}_{permeate}}{{AAV2\;Concentration}_{feed}}\right)\times 100$$

Each sample was measured in duplicate, and the average values were used to assess the efficiency of AAV2 recovery in the permeate after filtration.

### 2.9. Scanning Electron Microscopy (SEM) Preparation

Scanning electron microscopy (SEM) was employed to capture high-resolution images of pristine membranes. Small sections (approximately 1 cm^2^) of the tested membranes were cut and air-dried overnight to remove moisture. The membranes were non-conductive; thus, all samples were sputter-coated with a thin layer of platinum (~3 nm) to improve conductivity and image quality during SEM analysis. A ~3 nm coating of platinum was applied using a sputter coater under vacuum conditions. The samples were then mounted on an SEM stand using conductive carbon tape. Images were taken using the Helios NanoLab 660 SEM (FEI, Hillsboro, OR, USA).

### 2.10. Porosity

The porosity of the membranes was determined using an Anton Paar gas pycnometer (Anton Paar, Vernon Hills, IL, USA) to measure the volume of solid material. Before analysis, the membranes were cut into small circles of approximately 1.23 cm^2^. The membranes were weighed prior to analysis using a balance. The pycnometer was calibrated using a reference standard to ensure precise volume measurements. Five membrane circles were placed in the sample chamber of the gas pycnometer. Helium gas was used as the displacement medium due to its small molecular size and ability to penetrate the membrane pores. The pycnometer measured the volume of the solid portion of the membrane by calculating the displacement of the gas.

The porosity of the membranes was determined using the equation below:

$$\phi =\left(1-\frac{V_{pycnometer}}{V_{geometric}}\right)\times 100$$

where V_pycnometer_ is the volume of the membrane’s solid material obtained via the gas pycnometer, and V_geometric_ is the volume calculated by using the membrane’s physical dimensions. Each sample was measured in triplicate to ensure accuracy and reproducibility, and the average porosity values were reported.

### 2.11. Contact Angle

The contact angle of the membrane surfaces was measured to evaluate their hydrophilicity. The membrane samples, cut into small sections of approximately 1 cm^2^, were first cleaned with distilled water to remove any residual particles or contaminants and then dried for 24 h to remove moisture. The contact angle measurements were conducted using a KRÜSS drop shape analyzer (Matthews, NC, USA). Each membrane sample was placed on the stage, and a 10 µL droplet of distilled water was carefully deposited onto the membrane surface using a micropipette. Measurements were taken at ten different locations on each sample to ensure accuracy and reproducibility. The average initial contact angle was then calculated, providing insights into the hydrophilicity of the membrane surfaces. The contact angle over time is shown in Figure S10.

### 2.12. Statistical Analysis

A statistical analysis was applied to assess the significant differences between experimental conditions using a two-tailed Student’s *t*-test. Comparisons were made between parameters, such as recovery, turbidity, protein, and DNA reduction, under varying conditions (e.g., flow rates, filter materials, and salinity levels). The results are presented as mean ± standard deviation, with statistically significant differences marked in the figures by a single asterisk (*) for *p*-values < 0.05 and a double asterisk (**) for *p*-values < 0.01.

## 3. Results

### 3.1. Primary Clarification via Depth Filtration

#### 3.1.1. Depth Filter Characterization

Two commercial depth filters commonly used for clarification were analyzed for the clarification of mAAV2 crude harvest and real AAV2 crude harvest. The filters differed in their material composition, ionic capacity (tested via a counter-ionic displacement method), and pore size distribution, as detailed in Table 2.

Filter A consists of a single-layer structure with a relatively narrow pore size distribution. Its pore size transitions gradually throughout the thickness of the depth filter media. Conversely, Filter B features a four-layer structure with an abrupt pore size transition and a broader pore size distribution. The pore size distributions of the depth filters were obtained from manufacturer specifications and corroborated by data from previous studies [43]. To characterize the electrostatic binding of the depth filters, a counterionic displacement method was used where Filter A was determined to be 9.2 µeq/cm^2^ (1.8 µeq/cm^3^) and Filter B was 19.1 µeq/cm^2^ (3.7 µeq/cm^3^). Filter B’s design facilitates a combination of size-exclusion and adsorption mechanisms, with its enhanced ionic capacity enabling the effective removal of charged impurities like host cell DNA and proteins [16,21]. These differences in layer structure, pore size distribution, and ionic capacity provide a framework for investigating the relative contributions of physical sieving and adsorptive interactions to impurity removal and AAV2 recovery during clarification.

#### 3.1.2. Depth Filter Separation Mechanism Analysis

**Figure 1:**
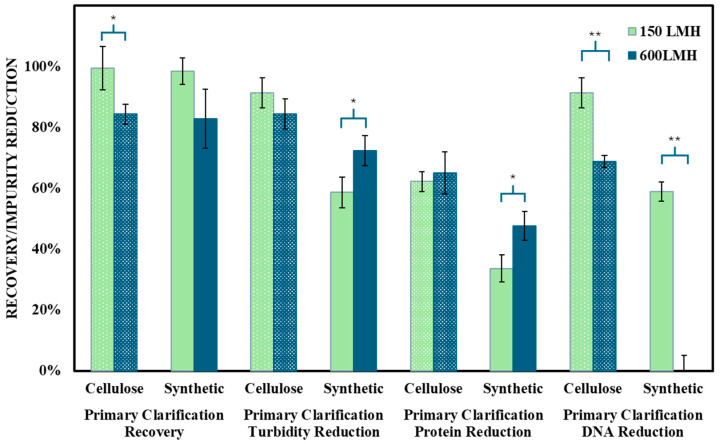
Comparison of model AAV2 recovery (%), turbidity, protein, and DNA reduction (%) between depth filters at two constant permeate flux rates (150 LMH and 600 LMH). Green bars represent 150 LMH, and blue bars represent 600 LMH. Solid bars correspond to the synthetic polyacrylic acid fiber filter, while patterned bars correspond to the cellulose filter. Results are presented as mean ± SD, with n = 3 for each condition. Two-tailed Student’s *t*-test analyzed statistical significance, where * *p* < 0.05 and ** *p* < 0.01. Statistically significant differences between depth filters at paired flow/permeate flux rates were observed for turbidity (**—150 LMH, *—600 LMH), protein (**—150 LMH, *—600 LMH), and DNA (**) reduction under both permeate flux rates.

The separation mechanisms of the depth filters were assessed under varying pump flow rates and permeate flux conditions to examine the interplay between impurity removal and mAAV2 recovery, as illustrated in Figure 1. This analysis highlights how flow rate impacts the balance between size exclusion and adsorptive behaviors, offering valuable insights into the filters’ performance dynamics.

For Filter A (cellulose-based), both recovery and DNA reduction were strongly influenced by pump flow rate/permeate flux. At a lower flux rate of 150 LMH, commonly employed during depth filtration processes, mAAV2 recovery exceeded 99%, achieving the desired target. Additionally, DNA reduction was remarkably effective, reaching over 90% without requiring endonuclease treatment. At a higher flux rate (thus, a lower residence time) of 600 LMH, which was hypothesized to decrease adsorptive interactions, a 15% reduction in mAAV2 recovery and a 22% decline in DNA clearance were observed, suggesting that the increased flux compromised the filter’s adsorptive efficiency for DNA.

In contrast, Filter B (synthetic-based) displayed notable differences in turbidity, protein, and DNA reduction based on the flux rate. At a lower flux rate of 150 LMH, turbidity and protein reduction were more than 15% lower than at the higher flux rate of 600 LMH. However, DNA reduction followed an inverse trend, showing negligible clearance at the higher flux rate, indicating a reduced capacity for DNA adsorption under these conditions.

These findings underscore the significant influence of flow rate on the performance of depth filters during AAV2 clarification. The lower flux rate (150 LMH) emerged as the optimal condition for further analysis, particularly with the real AAV2 crude harvest. This decision was driven by its ability to achieve target recovery rates (>99%) and substantial impurity removal, including effective DNA clearance and turbidity reduction, critical for downstream processing efficiency.

**Figure 2:**
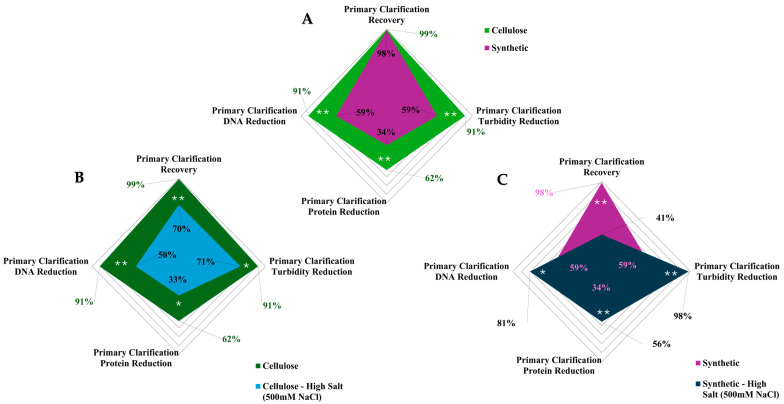
Spider charts comparing the performance of depth filters. Each chart represents key parameters: mAAV2 recovery, turbidity, protein, and DNA reduction. (**A**) overlays the results for both filter materials under standard salinity conditions, where green is Filter (**A**) (cellulose) and pink is Filter (**B**) (synthetic). (**B**) compares Filter (**A**) (cellulose) at both salinity conditions, and (**C**) compares Filter (**B**) (synthetic) at both salinity conditions. The results are mean ± SD (n = 3). Two-tailed Student’s *t*-test analyzed statistical significance, where * *p* < 0.05 and ** *p* < 0.01.

When comparing the depth filter materials side by side at the lower flux rate (150 LMH), notable differences in impurity reduction were observed across all metrics, as shown in Figure 2A. Filter A (cellulose-based), characterized by a single-layer design, gradual pore size transitions, and moderate ionic capacity, demonstrated a superior performance. It achieved mAAV2 recovery exceeding the target of >99% while also delivering significantly higher impurity reduction across DNA, protein, and turbidity metrics.

In contrast, Filter B (synthetic-based), which featured a four-layer structure, abrupt pore size transitions, and a high ionic capacity, exhibited considerably lower impurity reduction capabilities. These findings highlight the critical role that depth filter materials and morphological properties play in determining filtration efficiency and separation mechanisms. The differences in layer configuration, pore transition gradients, and filter media–nanoscale biologic interactions underscore the importance of tailoring filter design to optimize recovery and impurity clearance during viral vector clarification.

#### 3.1.3. Buffer Conductivity Analysis

Filter performance under high buffer conductivity parameters is critical for understanding effective purification methods for viral vector purification and has had limited prior assessment. Due to this, we tested two buffer conductivity/salinity parameters: standard buffer conductivity (~25 mS/cm; 150 mM NaCl), a commonly used condition for buffered-saline solutions, and high buffer conductivity (~320 mS/cm; 500 mM NaCl), shown to be optimal for solubility and stability for AAV2 [44]. Significant differences in depth filter performance were observed based on changes in buffer conductivity, shown in Figure 2B,C. Figure 2 presents a spider chart comparing the key filtration performance metrics between the mAAV2 crude harvest. Each axis represents a parameter (e.g., DNA removal, turbidity reduction), allowing for a holistic visualization of filtration performance differences. The chart highlights the performance differences between operating conditions and/or materials.

Both filters at high buffer conductivities showed an altered performance for all parameters analyzed. High buffer conductivity resulted in a >25% reduction in mAAV2 recovery for both filters tested. Filter A exhibited a 20% decrease in turbidity reduction, a 30% decrease in protein reduction, and a 40% decrease in DNA reduction. Filter B, on the other hand, exhibited a 39% increase in turbidity reduction, an 18% increase in protein reduction, and a 20% increase in DNA reduction.

**Figure 3:**
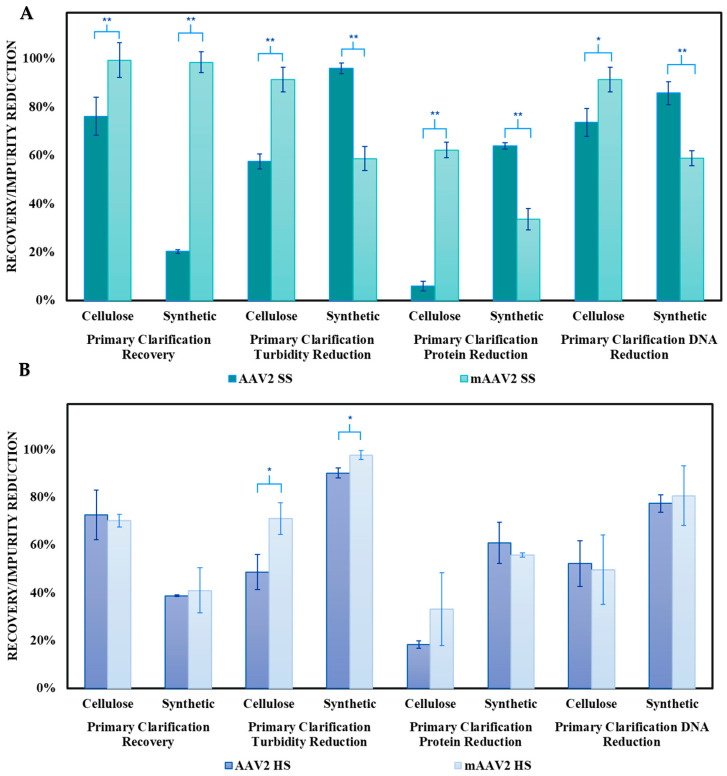
Performance comparison of Filter (**A**) (cellulose) and Filter (**B**) (synthetic) under standard conductivity/salinity (SS) ((**A**)—green) and high conductivity/salinity (HS) ((**B**)—purple) conditions for AAV2 (dark bars) and mAAV2 (light bars). The results are presented as the mean ± SD, with n = 3 (mAAV2) and n = 2 (AAV2) for each condition. Two-tailed Student’s *t*-test analyzed the statistical significance, where * *p* < 0.05 and ** *p* < 0.01.

These same conditions were then tested with real AAV2 crude harvest to evaluate the model’s predictability to assess depth filter performance. The data were replotted from Figure 2 in Figure 3 with the addition of the results from the real AAV2 crude harvest clarification.

Under standard buffer conductivity, the mAAV2 crude harvest exhibited significant differences in separation performance for both depth filters tested and for all parameters analyzed. However, under high buffer conductivity, the mAAV2 showed no significant differences compared to the real AAV2 crude harvest in the separation performance of all parameters, except for turbidity. This indicates the model system’s ability to match performance without significant differences from real AAV2 crude harvest systems for depth filtration.

Similar trends were observed in the real AAV2 system when evaluating the impact of buffer conductivity on depth filter performance. For Filter A, no significant differences were detected in AAV2 recovery or turbidity reduction across varying conductivity conditions. However, protein reduction improved by 12% at higher conductivity levels, likely due to enhanced interactions. Conversely, DNA reduction decreased by 22% under higher conductivity conditions, suggesting reduced adsorption capabilities for DNA at elevated salinity levels.

Filter B exhibited no significant changes in protein or DNA reduction under varying conductivities. However, a 20% increase in AAV2 recovery was observed at a higher conductivity, accompanied by a 6% decrease in turbidity reduction. These results underscore the differing sensitivities of the two filters to conductivity changes, reflecting the influence of their material properties and ionic capacities on separation performance.

### 3.2. Secondary Clarification via TFF with Microfiltration Membranes

#### 3.2.1. Microfiltration Membrane Characterization

Two commercially available microfiltration membranes, polyethersulfone (PES) and regenerated cellulose (RC), were tested to evaluate their suitability for secondary clarification following primary clarification with a cellulose-based depth filter. Both membranes featured a nominal pore size of 0.2 µm as indicated by their supplier and represented commonly used pore sizes and membrane materials for the separation of biologics [24]. However, characterization via surface SEM images revealed slight differences in pore size, with RC exhibiting marginally larger pores compared to PES, as shown in Table 3. Both membranes showed similar porosity and thickness, indicating comparable structural properties. However, key differences in the surface properties were identified: RC demonstrated a higher surface hydrophilicity, while PES exhibited a more negatively charged surface. These attributes were expected to influence performance, particularly in terms of fouling resistance, impurity reduction, and viral vector recovery.

#### 3.2.2. Performance of Microfiltration Membranes

**Figure 4:**
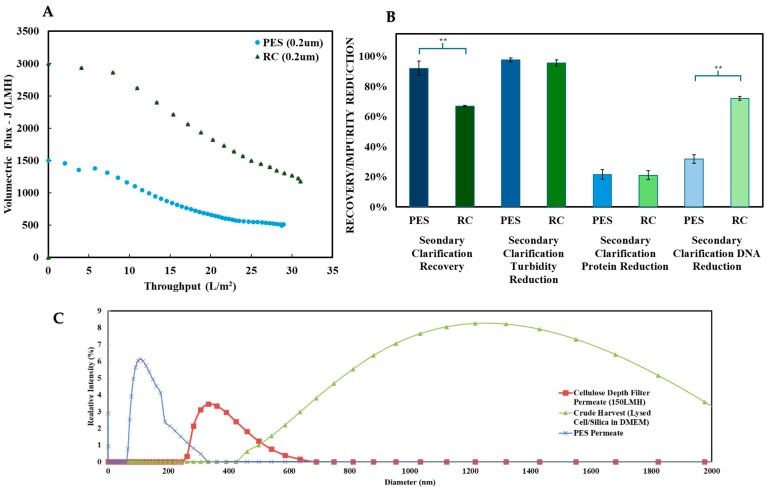
Comparison of the secondary clarification performance between the PES and RC membranes conducted at a constant pressure of 1 bar. The figure presents (**A**)—flux versus throughput for PES (blue—circle) and RC (green—triangle) on the left, and (**B**)—mAAV2 recovery, turbidity reduction, protein reduction, and DNA reduction. (**C**)—Dynamic light scattering (DLS) analysis of the mAAV2 feed and permeates obtained from the Filter A and PES membrane. These results depict the size distribution profiles for the feed (green), the permeate from the depth filter (red), and the permeate from the membrane (blue). These results illustrate the effectiveness of the filtration processes in reducing the size of particulates, providing insights into the removal efficiency of impurities and the recovery of model AAV2 particles during purification. The results are presented as the mean ± SD (n = 3). Two-tailed Student’s *t*-test analyzed statistical significance, where ** *p* < 0.01.

The performances of the PES and RC membranes were evaluated as secondary clarification steps following primary clarification with Filter A (cellulose-based) under optimal conditions: standard conductivity (150 mM NaCl) and low flux (150 LMH), as identified in Section 3.1. Figure 4 presents flux decline as a function of throughput, along with mAAV2 recovery and impurity reductions (turbidity, protein, and DNA) for both membranes.

Both membranes exhibited noticeable flux declines, with PES showing a 66% reduction and RC showing a slightly lower 61% reduction. Significant differences were observed in mAAV2 recovery and DNA reduction between the two membranes. PES outperformed RC in mAAV2 recovery, achieving >95%, which was a 30% improvement over RC. Conversely, RC exhibited superior DNA reduction, achieving a 78% decrease—a 40% increase compared to PES. For turbidity and protein reduction, no significant differences were noted between the two membranes, with both achieving >95% turbidity reduction and approximately 20% protein reduction.

**Figure 5:**
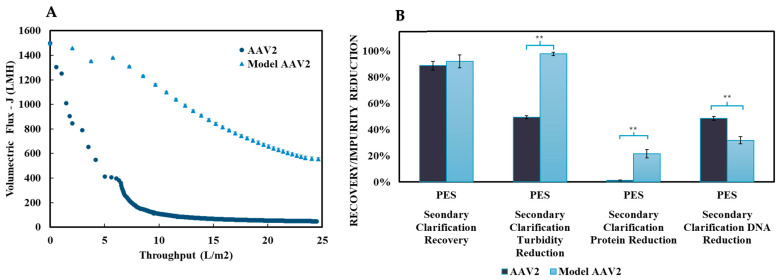
Comparison of secondary clarification results between mAAV2 crude harvest (light blue triangles) and real AAV2 crude harvest (dark blue circles). The figure presents (**A**)—flux versus throughput at a constant pressure of 1 bar with the PES membrane for mAAV2 (light blue—triangles) and AAV2 (dark blue—circles) secondary clarification, and (**B**)—mAAV2 recovery, turbidity reduction, protein reduction, and DNA reduction. Data are presented as the mean ± SD (n = 3 (model AAV2); n = 2 (AAV2)). Two-tailed Student’s *t*-test analyzed statistical significance, ** *p* < 0.01.

To assess the model system’s predictive capability, the PES membrane, which demonstrated optimal mAAV2 recovery, was tested for secondary clarification with actual AAV2 crude harvest following primary clarification with Filter A. Figure 5 compares the PES membrane’s flux decline, recovery, and impurity reduction performance between the mAAV2 model system and the actual AAV2 system.

Significant differences were observed in impurity reductions between the model and actual AAV2 systems, but no significant differences were observed in AAV2 recovery, indicating that the model system accurately predicted the membrane throughput performance. However, flux decline during secondary clarification showed notable differences, most likely attributed to the increased fouling discussed in Section 3.2.4. While the model system exhibited a 66% reduction in flux, the actual AAV2 system experienced a much steeper flux decline, reaching a 96% reduction, resulting in a final flux of ~50 LMH.

#### 3.2.3. Global Clarification

The global clarification, the effectiveness of the combined primary and secondary clarification, performance, and the influence of primary clarification on secondary membrane efficiency were evaluated for both the PES and RC membranes, as depicted in Table 4. Comparing the two membranes, PES demonstrated a superior recovery at 87%, while RC achieved 63% under standard conditions. Both membranes performed exceptionally in turbidity reduction, achieving >99% (<5 NTU), with no significant differences observed. Protein reduction was equivalent for both membranes at 78%, while DNA reduction without endonuclease treatment reached 98% for RC (resulting in ~5 ng/mL residual DNA, compliant with FDA guidelines of <10 ng/dose [46,47]) and 94% for PES (resulting in ~15 ng/mL residual DNA).

**Figure 6:**
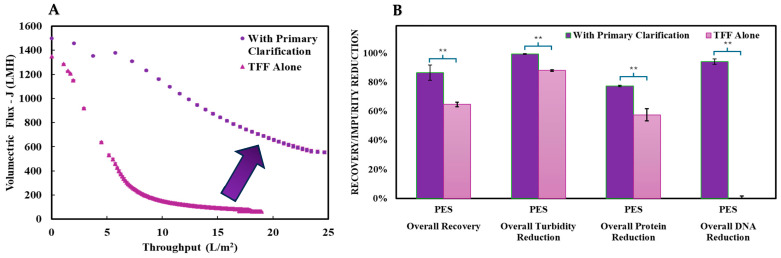
(**A**) Comparison of the impacts the addition of the primary clarification via depth filtration had on the secondary clarification results between the mAAV2 crude harvest with the primary clarification (purple—circles) and without (pink—triangles). (**B**) The figure presents mAAV2 recovery, turbidity reduction, protein reduction, DNA reduction, and membrane flux for both systems using the PES membrane at a constant pressure of 1 bar. Data are presented as the mean ± SD (n = 3). Two-tailed Student’s *t*-test analyzed statistical significance, where ** *p* < 0.01.

The addition of primary clarification via Filter A significantly enhanced the performance of the PES membrane, as shown in Figure 6. Recovery increased by 20%, reaching 87%, with a 12% improvement in turbidity reduction. Protein reduction also rose by 20%, reflecting the complementary role of the depth filter in removing larger impurities upstream. Most notably, flux decline was mitigated dramatically, with a 100-fold improvement in throughput at 18 L/m^2^, increasing from 60 LMH to 600 LMH. These enhancements demonstrate the critical role of primary clarification in optimizing membrane performance during secondary clarification.

For the PES membrane, the real AAV2 system exhibited a reduced overall performance compared to the model system. Recovery dropped to 60%, turbidity reduction decreased to 90% (<20 NTU), protein reduction fell to 23%, and DNA achieved a reduction of 85% without endonuclease treatment. The diminished performance in the real system can be attributed to the presence of higher levels of residual protein and DNA, likely due to the hindered performance during primary clarification. This underscores the complexity of real-world applications and the importance of designing upstream and downstream platforms in parallel in order to optimize product titer and purification.

#### 3.2.4. Fouling Analysis of mAAV2 and Real AAV2 Crude Harvest During Secondary Clarification

The mechanisms of fouling during the secondary clarification of both the model (mAAV2) and real (AAV2 crude harvest) systems using the PES membrane were investigated using standard fouling models, as shown in Table 5 [48,49,50]. These models offer insights into the dominant fouling behaviors by characterizing the interactions between impurities and the membrane surface during filtration.

**Figure 7:**
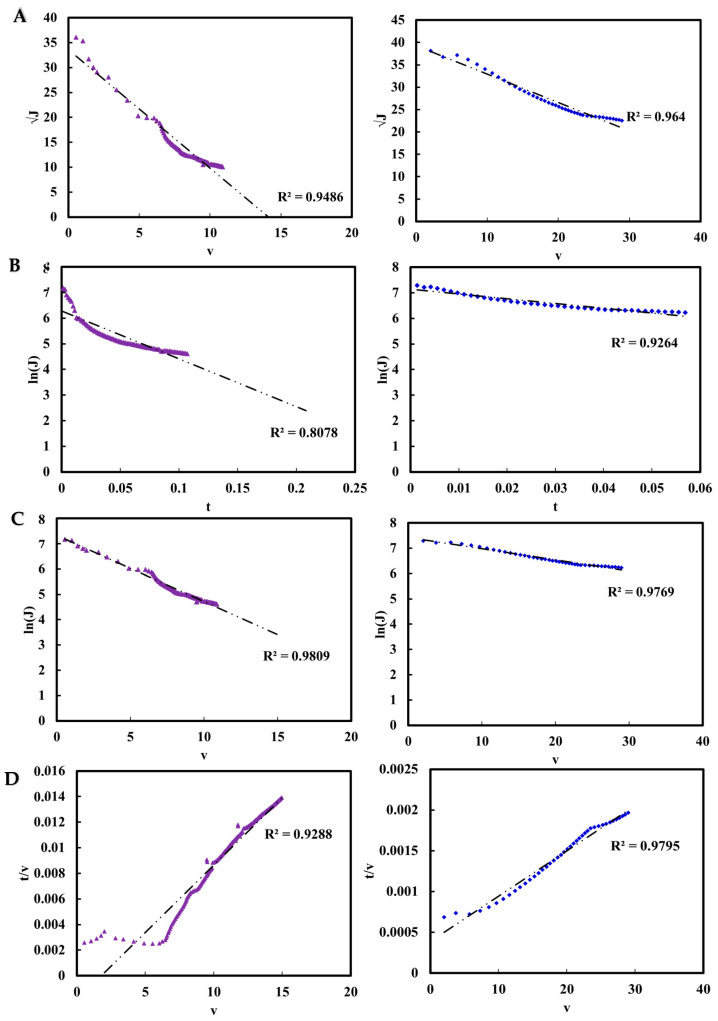
Flux decline analysis during the secondary clarification of mAAV2 (right—blue diamonds) and real AAV2 (left—purple triangles) crude harvest using fouling models: (**A**) standard blocking, (**B**) complete blocking, (**C**) intermediate blocking, and (**D**) cake layer formation. Each plot represents the experimental flux decline fitted to the corresponding fouling model.

For the model system, the flux decline closely matched the cake layer formation model throughout the filtration process, as depicted in Figure 7. This indicates that fouling was primarily driven by the accumulation of particles and impurities on the membrane surface, forming a dense fouling layer that impeded fluid flow. The fouling parameter values derived for the model system (K_B_, K_S_, K_I_, and K_c_) were substantially lower compared to the real system, suggesting that the simplified feed composition of the model system imposed fouling and resistance during filtration.

Regarding the real AAV2 crude harvest, the fouling behavior initially aligned best with the intermediate blocking model. This reflected a partial pore obstruction due to the smaller impurities, such as fragmented proteins and DNA, interacting with the membrane surface or partially blocking the pore openings. As filtration progressed, the fouling mechanism shifted to a cake layer formation, consistent with the accumulation of aggregated proteins and cell debris on the membrane surface.

The transition in the real system highlighted the dynamic nature of fouling mechanisms under complex feed conditions. Additionally, the fouling parameter values, shown in Table 6, for the real system were consistently higher than those of the model system across all models, indicating a more severe fouling impact.

The disparity in fouling mechanisms and parameter values between the model and real systems underscored the influence of feed composition on fouling dynamics. While the model system provided a simplified framework for evaluating surface fouling resistance, the real system demonstrated a more complex and evolving fouling profile.

Understanding the fouling behavior of the PES membrane during secondary clarification provides critical insights into the challenges of processing complex biological feed systems. These findings emphasize the need for tailored pre-treatment and membrane surface modifications to mitigate fouling and optimize viral vector purification processes.

## 4. Discussion

The observed separation mechanisms underscore the complex interplay between material properties, filter morphology, and operational parameters, such as flow rate, in the depth filtration of AAV2. Both size-exclusion and adsorptive mechanisms were influential in impurity removal; however, their relative contributions were contingent on the design and operational conditions of the filters.

The influence of flow rate on separation efficiency was evident in the performance differences between Filter A (cellulose-based) and Filter B (synthetic-based). For Filter A, lower flux rates (150 LMH) facilitated extended interactions between impurities and the filter matrix, enhancing the adsorptive and size-exclusion efficiency. This was evident with an over 90% DNA reduction without endonuclease treatment, consistent with the literature reporting that slower flux rates improve impurity retention by increasing contact time [14,21]. Conversely, higher flux rates (600 LMH) reduced the impurity–filter interaction time, limiting opportunities for DNA–membrane interactions and increasing the likelihood of DNA passing through the filter. Filter B’s performance was more variable, with turbidity and protein reductions improving at higher flux rates, yet DNA removal remained suboptimal. This variability suggests that Filter B’s high ionic capacity and abrupt pore transitions may preferentially adsorb specific impurities at lower flux rates, but fail to achieve consistent, broad-spectrum removal.

Material composition and ionic interactions also significantly influenced filter performance. For Filter A, the moderate ionic capacity likely supported DNA binding through electrostatic interactions. However, as conductivity/ionic strength increased (via increased NaCl concentration), the higher ionic strength screened these electrostatic interactions, reducing the effectiveness of charge-based adsorption and leading to lower DNA retention [51]. Additionally, increased ionic strength can alter the conformation of DNA molecules, reducing their effective charge density and further diminishing filter–DNA interactions [51,52]. This is corroborated by studies in chromatography and membrane separations, which demonstrate that high ionic strength solutions disrupt the binding of charged species [11,14,16,53]. Filter B, with its synthetic material and high ionic capacity, exhibited more stable impurity removal across conductivity conditions.

The stability of AAV2 particles in high-conductivity environments, as reported in the literature, also played a role. Enhanced stability likely reduced particle aggregation and adsorption, contributing to a higher recovery in Filter B under these conditions [44]. However, this stability could shift the dynamics of impurity interactions, necessitating tailored process conditions for effective separation.

In the context of secondary clarification, the material properties of PES and RC membranes further highlighted the impact of surface charge and hydrophilicity. The higher negative charge of the PES membrane minimized the non-specific adsorption of AAV2 particles, preserving recovery. In contrast, the less negatively charged RC membrane showed greater DNA reduction, likely due to the stronger electrostatic interactions with negatively charged DNA molecules.

Fouling behavior in the real and model systems emphasized the role of feed composition and operational dynamics in membrane performance. The real AAV2 system exhibited a transition from intermediate blocking to cake layer formation, driven by smaller impurities initially obstructing pore entrances, followed by the accumulation of larger protein aggregates and cellular debris. The model system being dominated by cake layer formation from the outset reflects its simpler composition and the absence of smaller impurities, highlighted by the superior performance of the depth filters for the model system. While model systems are invaluable for controlled studies, their limited ability to replicate the complexity of real feedstocks limits their predictive capability for fouling dynamics in operational settings.

The higher fouling parameter values observed in the real system highlight the severity of fouling due to an increased impurity load and diversity. The transition from intermediate blocking to cake layer formation in the real AAV2 system suggests that initial pore blockage by smaller impurities gives way to the accumulation of a dense fouling layer over time. In contrast, the model system predominantly exhibited cake fouling from the outset, indicating that its simplified impurity profile reduces the smaller biomolecular interactions seen in real systems. The transition to cake layer formation has practical implications for process optimization. Strategies such as pre-filtration or membrane functionalization to mitigate fouling during the intermediate blocking stage may delay severe fouling associated with cake layer formation. Additionally, optimizing operational protocols, such as alternating flow regimes or developing fouling-resistant membranes, could further enhance performance.

Our study demonstrates significant practical advancements in the biomanufacturing of AAV2 viral vectors. By optimizing depth and membrane filtration strategies, we achieved high viral vector recovery rates exceeding 90%, surpassing typical industry recoveries that often range from 40% to 80%. This enhancement not only improves the product yield but also reduces operating times by streamlining the purification process. Moreover, our method effectively reduces impurities without the need for endonuclease treatment, leading to significant cost savings (estimated >100,000 USD per 500 L batch) [20]. Traditional processes often rely on endonucleases to degrade host cell DNA, which adds complexity and expenses to the manufacturing process. By eliminating this step, we simplify the workflow and reduce costs, aligning with the industry goals for increased efficiency and scalability. Furthermore, the use of a synthetic model system allows for rapid process optimization, reducing the need for costly viral vector materials during early-stage development, as well as the indicated parameters needed to fully mimic the actual AAVs’ throughput with the model system. These findings align with the industry goals of improving scalability and reducing bioprocessing costs.

## 5. Conclusions

This study presents a detailed evaluation of depth and secondary membrane filtration for AAV2 recovery and purification, offering novel insights into the interplay of material properties, operational conditions, and feed composition in viral vector processing. The objectives were to (1) assess the performance of depth filters for primary clarification under varying flow rates, conductivity conditions, and material properties without the use of endonucleases; (2) analyze secondary filtration with PES and RC membranes in terms of recovery, impurity removal, and fouling mechanisms; and (3) compare a synthetic nanoparticle-based model system with real AAV2 crude harvest for evaluating filtration techniques.

The evaluation of depth filters during primary clarification revealed the significant effects of flow rate and buffer conductivity on impurity removal. At high flow rates (600 LMH), the depth filter’s capacity for adsorptive and electrostatic interactions was diminished, reducing the DNA removal efficiency as size-exclusion became the dominant mechanism. Conversely, lower flow rates (150 LMH) enabled enhanced adsorptive interactions, achieving DNA reductions exceeding 90% for the model system without enzymatic treatment under optimized conditions. These findings emphasize the method’s potential for cost and time savings of an estimated 100,000 USD at large-scale production (500 L) [20]. High-conductivity conditions further impacted depth filter performance by screening electrostatic interactions, particularly for lower charge cellulose-based filters, aligning with established membrane and chromatographic separation principles. Notably, the model system closely matched the depth filter performance observed in the real system under high-conductivity conditions, demonstrating its utility as a predictive tool for initial evaluations.

Secondary clarification using the PES and RC membranes demonstrated material-dependent trade-offs between recovery and impurity removal. The PES membrane achieved exceptional AAV2 recovery rates exceeding 90%, attributed to its highly negative surface charge, which minimized nonspecific adsorption. In contrast, the RC membrane excelled in DNA reduction (>80%) due to its moderate surface charge and enhanced hydrophilicity, which promoted stronger adsorptive interactions. Importantly, the integration of the primary clarification step significantly improved the PES membrane’s final flux, increasing from ~60 LMH without depth filtration to ~600 LMH, thereby enhancing the process efficiency.

Fouling analysis underscored the complexity and severity of the challenges associated with real AAV2 systems. While the model system predominantly exhibited cake layer formation, the real system transitioned from intermediate blocking to severe cake layer fouling due to its more diverse impurity profile. This difference highlights the importance of validating filtration processes with real feedstock to account for the operational realities and to refine fouling mitigation strategies. While the model system effectively predicted filtration performance at a high conductivity, its limitations for fouling analysis should be considered. Future applications should incorporate charge-modified nanoparticles or additional impurity components to improve predictive accuracy. Furthermore, future research should focus on surface-functionalized membranes to enhance recovery and impurity removal while mitigating fouling.

This work represents a significant step forward in addressing the challenges of AAV2 purification, providing actionable solutions that reduce costs and processing times, and enhance recovery while improving membrane performance. By bridging gaps in the literature and offering a robust framework for evaluating filtration systems, this study supports the development of scalable and efficient processes for biologics production, ultimately advancing accessibility to gene therapies.

## Figures and Tables

**Table 1:** Comparison of mAAV2 and AAV2 properties.

	Size (nm)	Density (g/cm^3^)	Zeta Potential at Operating pH (~7.5) in Phosphate Buffer Solution (PBS) (1X)
AAV2	18–26	1.39 [7]	−15 ± 5 [39,40,41]
mAAV2	21.5 ± 0.13 *	~2	−19.4 ± 1 [34]

* Indicated by manufacturer (SuperiorSilica, Chandler, AZ, USA).

**Table 2:** Properties of depth filters chosen for primary clarification of AAV2 crude harvest.

	Material Composition	Pore Distribution (µm)	Layers	Pore Size Transition	Ionic Capacity
Filter A	Cellulose	1 to 0.6 *	Single *	Gradual *	Moderate (9.2 µeq/cm^2^)
Filter B	Polyacrylic acid fibers	9 to 0.6 *	Four *	Abrupt *	High (19.1 µeq/cm^2^)

* Indicated by manufacturer (MilliporeSigma, Saint Louis, MO, USA) and corroborated by [43].

**Table 3:** Microfiltration membrane properties.

	PES	RC
Membrane Material	Polyether sulfone	Regenerated cellulose
Pore Size (µm)	0.25 ± 0.06	0.29 ± 0.12
Thickness (µm)	165 ± 15	160 ± 12
Contact Angle (°)	41.1 ± 8.2	20.3 ± 1.6
Porosity (%)	61.0 ± 4.6	57.4 ± 1.2
Zeta Potential (mV)	−15–25 [45]	−5–10 [45]

Note: zeta potential is documented at a of pH~7.

**Table 4:** Global clarification of mAAV2 (blue) and AAV2 (gray) crude harvest post-primary clarification via Filter A (cellulose) and secondary clarification via microfiltration membranes.

	AAV Vector Titer ^a^ (capsids/mL)	AAV Vector Recovery (%)	Turbidity (NTU) ^b^	Turbidity Reduction (%)	HCP (mg/mL) ^c^	HCP Reduction (%)	DNA (ng/mL) ^d^	DNA Reduction
Model AAV2 Crude Harvest	5 × 10^14^ ± 3.5 ×10^11^	N/A	230 ± 13.52	N/A	5.9 ± 0.22	N/A	270.5 ± 15.4	N/A
Overall Clarification PES	4.34 × 10^14^ ± 2.28×10^13^	86.8 ± 5.3	0.99 ± 0.3	99.6 ± 0.3	1.32 ± 0.7	77.6 ± 0.6	15.34 ± 2.8	94.3 ± 1.8
Overall Clarification RC	3.16 × 10^14^ ± 6.39×10^12^	63.1 ± 2.0	1.85 ± 0.5	99.2 ± 0.3	1.29 ± 0.4	78.0 ± 0.3	5.27 ± 0.8	98.0 ± 0.2
AAV2 Crude Harvest	1.64 × 10^12^ ± 6.45×10^8^	N/A	187.13 ± 3.38	N/A	4.33 ± 0.48	N/A	3873.4 ± 22.1	N/A
Overall Clarification	9.91 × 10^11^ ± 2.21 × 10^10^	60.3 ± 2.2	17.91 ± 0.8	89.4 ± 0.4	3.19 ± 0.1	22.8 ± 0.2	589.1 ± 0.68	84.8 ± 1.1

^a^ ELISA; ^b^ turbidity meter; ^c^ Bradford assay; ^d^ fluorescence assay (bisbenzimide).

**Table 5:** Fouling models for constant pressure membrane filtration.

Standard Blocking	Complete Blocking	Intermediate Blocking	Cake Layer
$\sqrt{J}=\sqrt{J_{o}}-\frac{K_{s}\sqrt{J_{o}}}{2}v$	$ln(J)=ln(J_{o})-K_{B}t$	$\ln\left(J\right)=ln(J_{o})-K_{I}t$	$\frac{t}{v}=\frac{1}{J_{o}}+K_{C}v$
(a) 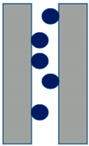	(b) 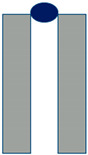	(c) 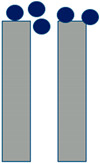	(d) 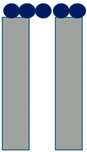

Note: J_o_ is the initial flux; K_B_, K_S_, K_I_, and K_c_ are the fouling constants; v is the throughput; and t is the time. Adapted from [48,49,50].

**Table 6:** Fouling constant comparison for mAAV2 and AAV2 crude harvest fouling analysis.

	Standard Blocking Parameter (K_S_)	Complete Blocking Parameter (K_B_)	Intermediate Blocking Parameter (K_I_)	Cake Layer Parameter (K_C_)
mAAV2	−0.63	−18.5	−0.04	6 × 10^−5^
AAV2	−2.38	−18.67	−0.26	0.0011

## Data Availability

The raw research data supporting the conclusions of this article will be made available by the authors on request.

## Supplementary Materials

The following supporting information can be downloaded at: https://www.mdpi.com/article/10.3390/nano15040310/s1, Figure S1: Constant flux of depth filters; Figure S2: Pure water flux of membranes; Figure S3: SEM of pristine membranes; Figure S4: TFF experimental setup; Figure S5: DLS analysis of mAAV2 crude harvest; Figure S6: BSA concentration curve; Figure S7: DNA concentration curve; Figure S8: mAAV2 concentration curve; Figure S9: AAV2 sandwich ELISA concentration curve; Figure S10: Contact angle of membranes over time; Figure S11: NaCl concentration curve. Supplemental Section S2: Materials and Methods.

## Abbreviations

The following abbreviations are used in this manuscript:

AAV2	Adeno-associated virus serotype 2
mAAV2	Model adeno-associated virus serotype 2
PES	Polyethersulfone
RC	Regenerated cellulose
LMH	Liters per square meter per hour (unit of flux)
NTU	Nephelometric Turbidity Unit
DNA	Deoxyribonucleic Acid
Ks	Standard blocking constant
Kb	Complete blocking constant
Ki	Intermediate blocking constant
Kc	Cake layer formation constant
TFF	Tangential flow filtration
FDA	Food and Drug Administration
DMEM	Dulbecco’s Modified Eagle Medium
PBS	Phosphate buffer solution
