# Supplementary material for: Membrane Filtration of Nanoscale Biomaterials: Model System and Membrane Performance Evaluation for AAV2 Viral Vector Clarification and Recovery
Source: Nanomaterials (Basel). 2025 Feb 18;15(4):310. doi: 10.3390/nano15040310 (PMC11858591; doi:10.3390/nano15040310)
Supplement: Supplementary file 1 [file nanomaterials-15-00310-s001.zip › nanomaterials-3453095-supplementary.pdf]

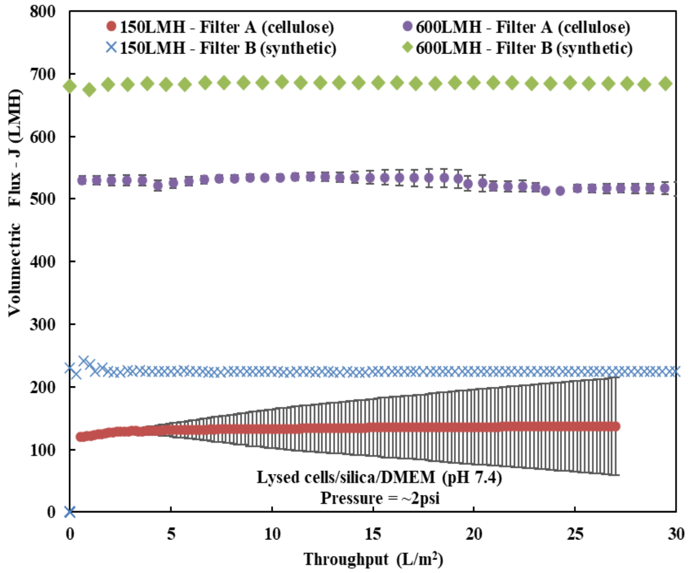

Figure S1. Constant flux versus throughput for Filter A (cellulose) and Filter B (polyacrylic) depth filters at tested flow rates. Data are shown as mean ± SD (n=3). This comparison highlights differences in flux stability and throughput capacity between the two depth filters under varying operational flow conditions.

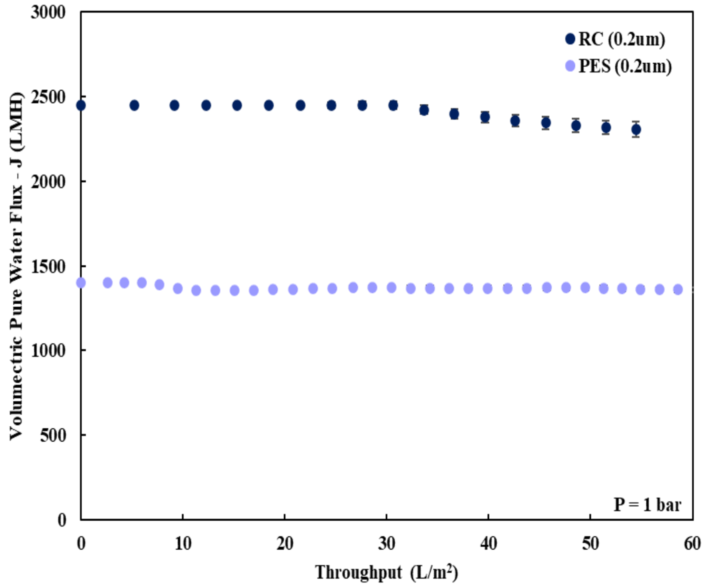

Figure S2. Pure water flux versus throughput for the PES and RC membranes tested during secondary clarification. Data are presented as mean ± SD (n=3).

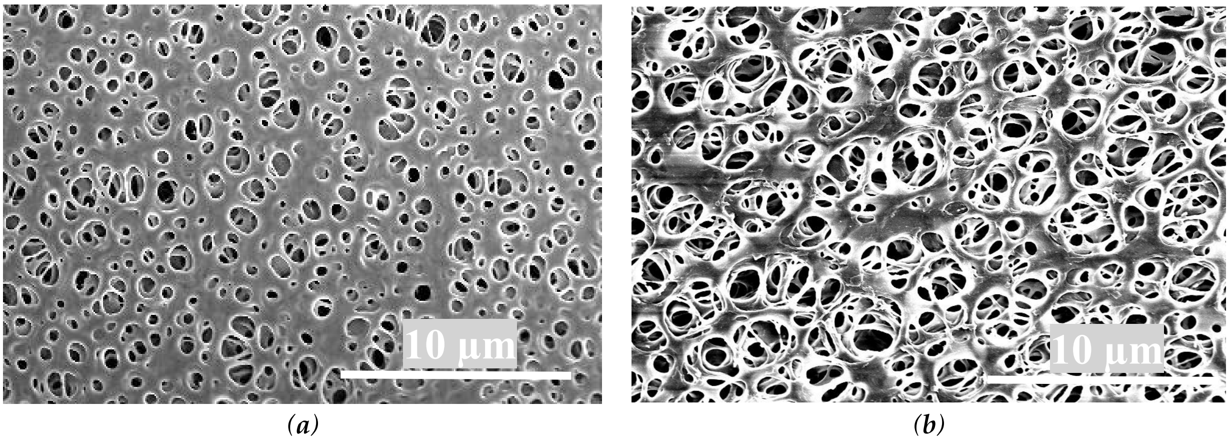

Figure S3. Scanning electron microscopy (SEM) images of the surface morphology of pristine membranes: (a) PES membrane and (b) RC membrane.

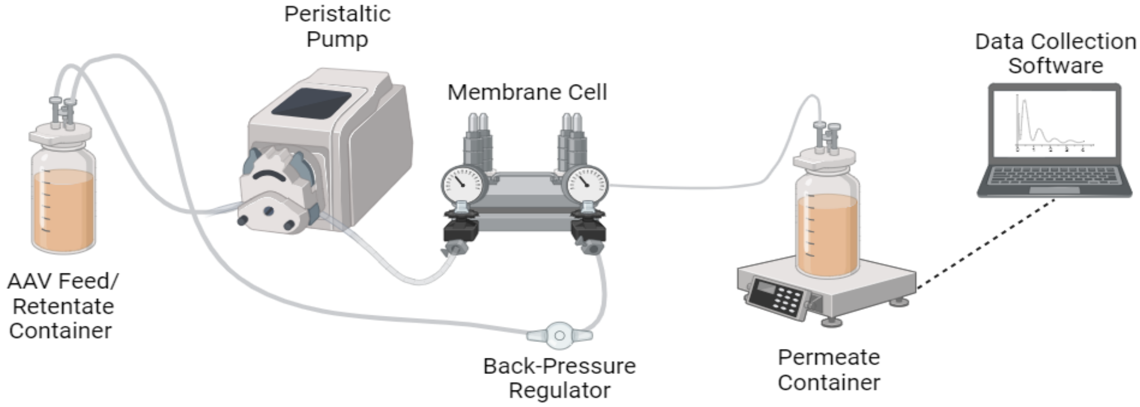

Figure S4. Tangential flow filtration (TFF) experimental setup.

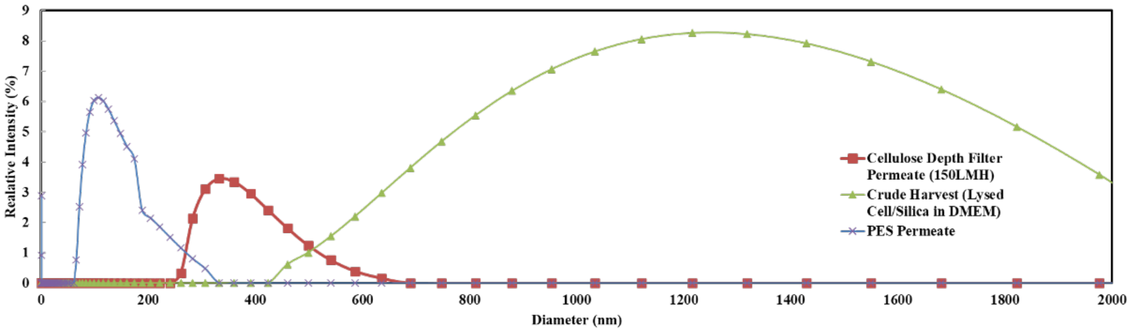

Figure S5. Dynamic light scattering (DLS) analysis of the model system feed and permeates obtained from the depth filter and membrane filtration. The plots depict the size distribution profiles for the feed (green), the permeate from the depth filter (red), and the permeate from the membrane (blue). These results illustrate the effectiveness of the filtration processes in reducing the size of particulates, providing insights into the efficiency of impurity removal and the recovery of model AAV2 particles during purification.

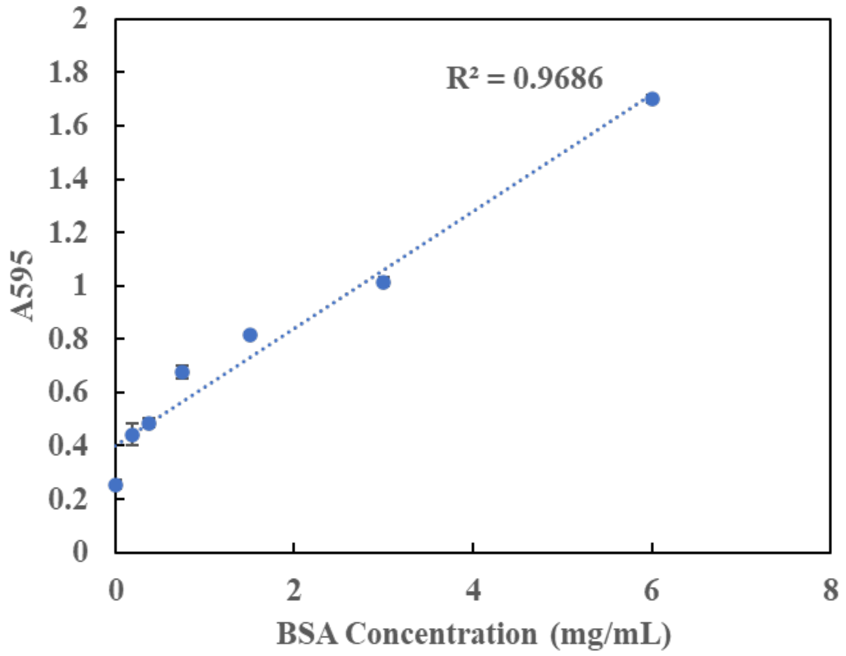

Figure S6. BSA concentration curve from 0 – 6 mg/mL.

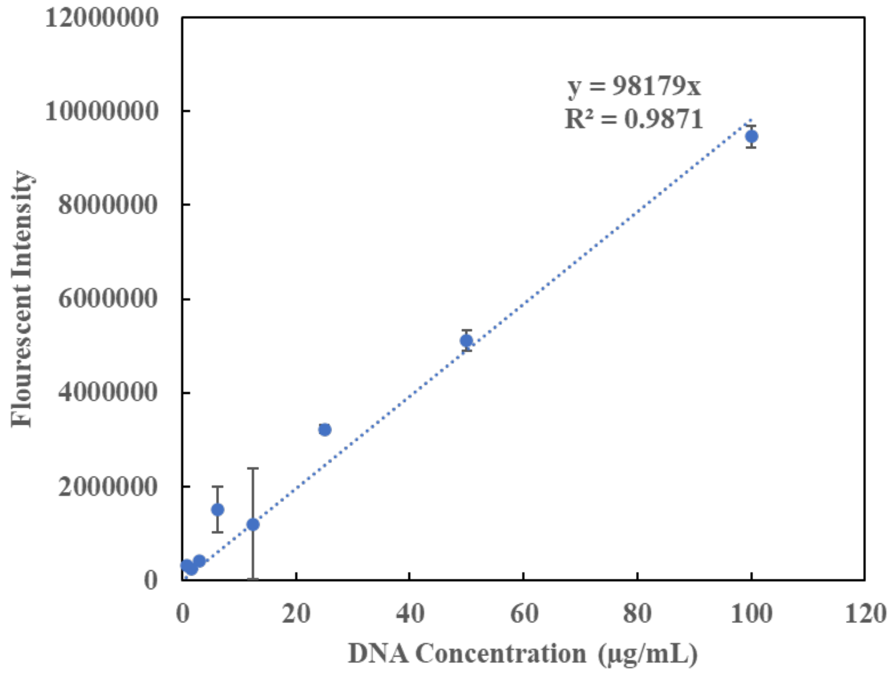

Figure S7. DNA concentration curve from 0 – 100 μg/mL.

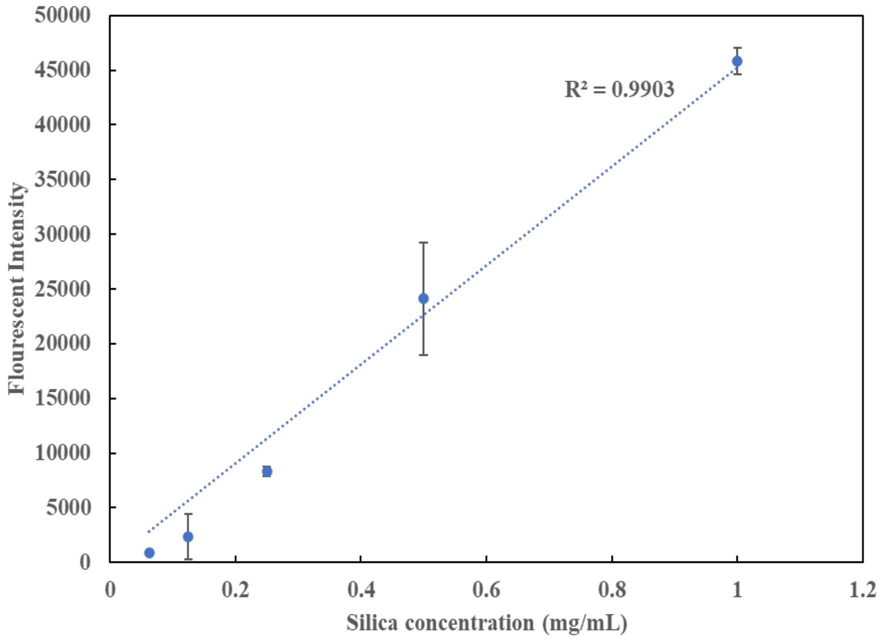

Figure S8. Green fluorescent silica nanoparticle concentration curve (model AAV2) from 0-1 mg/mL.

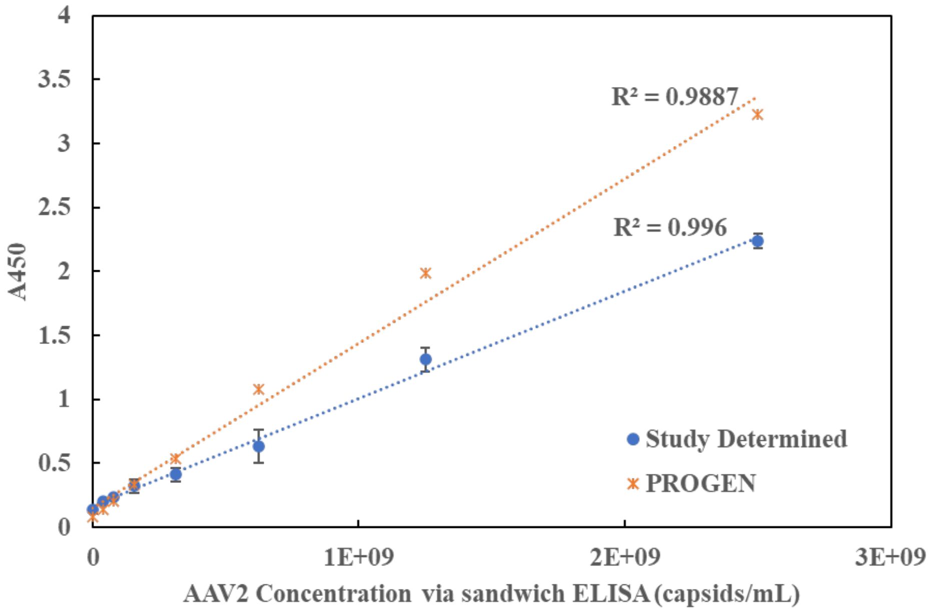

Figure S9. AAV2 concentration curve via the PROGEN sandwich AAV2 ELISA kit. Both the given concentration curve via the standard (PROGEN) and the study-determined concentration via the standard are provided.

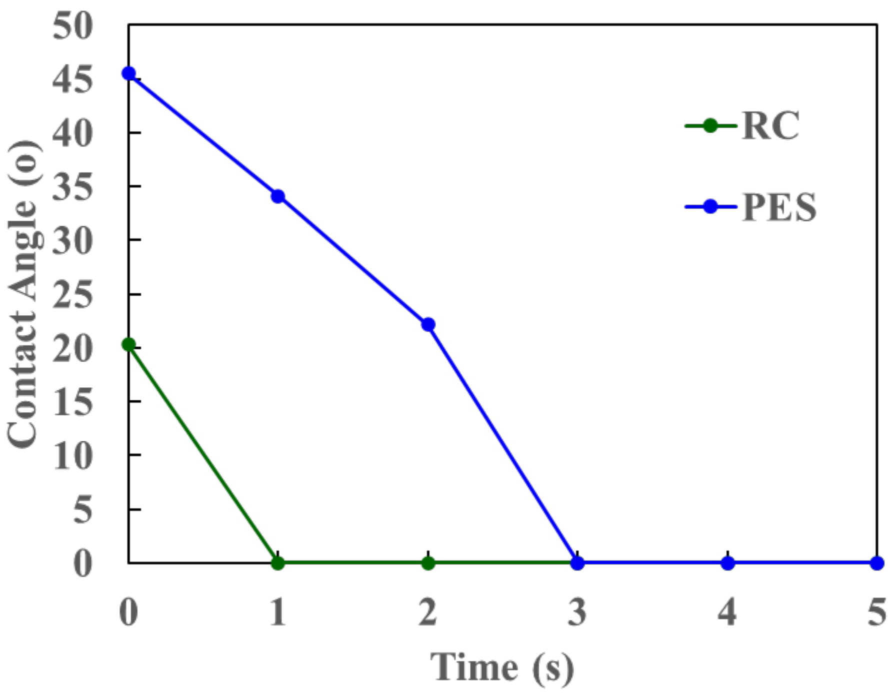

Figure S10. Contact angle decay for the two microfiltration membranes tested.

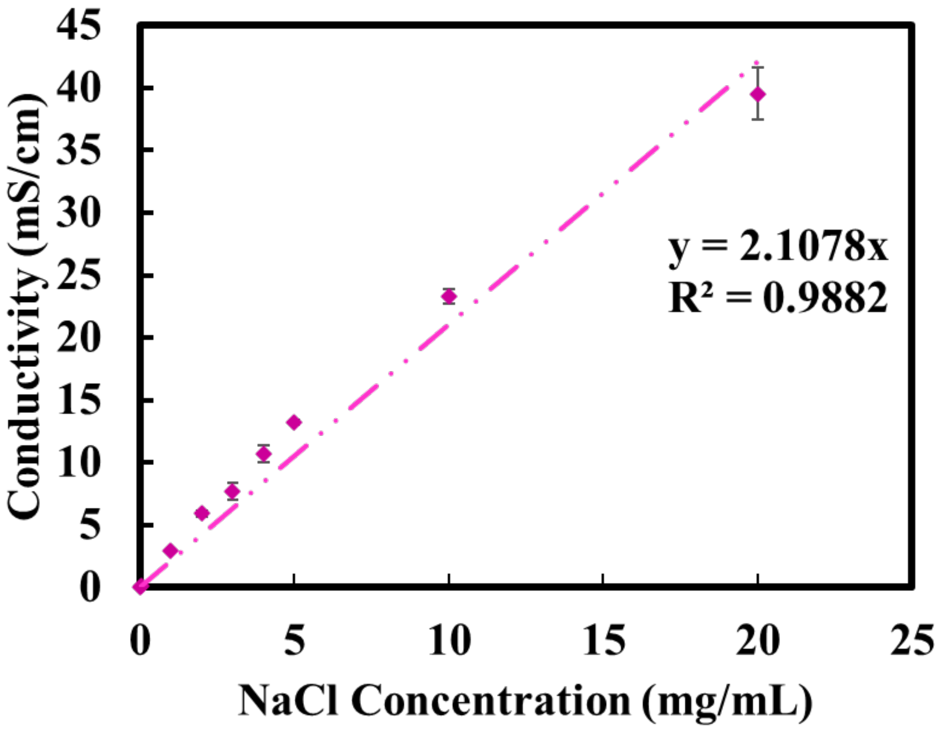

Figure S11. NaCl concentration curve determined via conductivity probe from 0 – 20 mg/mL.

S2. Materials and Methods Supplemental

S2.8.1. Turbidity

The turbidity of both the feed and permeate solutions was measured using a calibrated Apera Instruments TN400 portable turbidity meter (ISO 7027). Before each use, the meter was calibrated using turbidity standards ranging from 0 to 800 NTU. Approximately 25 mL of each sample was transferred into a clean testing vial. The exterior of the

vial was thoroughly cleaned with a Kimwipe, and silicon oil was applied if necessary to eliminate any smudges or debris that could interfere with the measurement. The vial was then placed in the turbidity meter, and the turbidity of the sample was recorded. Each sample was measured at least three times, with gentle mixing between readings to prevent the settling of particulates.

S2.8.2. Dynamic Light Scattering

The size distribution of the particles, in both the feed and permeate solutions, was determined using dynamic light scattering (DLS) analysis. A volume of 1 mL from each sample was transferred into a disposable cuvette, ensuring the absence of bubbles. The cuvettes were then carefully cleaned with a lint-free Kimwipe to remove any dust or smudges on the exterior. Measurements were conducted using an Anton Paar DLS particle analyzer. Each sample was analyzed at room temperature, and measurements were conducted in triplicate. Between each measurement, the sample was briefly vortexed to prevent particle settling. The particle size was determined based on the Stokes–Einstein equation. The particle size distribution was reported as the mean hydrodynamic diameter, with the polydispersity index (PDI) used to assess the distribution's uniformity. The DLS analysis of the feeds and permeates are shown in S5.

S2.8.3. Protein Quantification

The protein concentration of both the feed and permeate solutions was determined using a Bradford protein assay (ThermoFisher, 23200). A standard curve (S6) was first generated using bovine serum albumin (BSA) standards supplied with a kit, with known concentrations ranging from 0 to 6 mg/mL. The Bradford reagent was prepared according to the manufacturer's instructions, and 200 μL of the reagent was added to each well of a 96-well plate containing 50 μL of either the sample or standard. The plate was incubated at room temperature for 5-10 minutes. Absorbance was then measured at 595 nm using a BioTeck Synergy hybrid or SpectraMax iD3 microplate reader. The protein concentration of the feed and permeate samples was determined by comparing the absorbance values to the BSA standard curve.

The percentage of protein reduction was calculated using the equation below:

$$\text{Protein Reduction (\%)} = \left(\frac{\text{Protein Concentration}_{\text{feed}} - \text{Protein Concentratoin}_{\text{permeate}}}{\text{Protein Concentration}_{\text{feed}}}\right) \times 100$$

This analysis allowed for the quantification of protein removal during the filtration process, with each sample analyzed in triplicate.

S2.8.4. DNA Quantification

The DNA concentration of both the feed and permeate solutions was determined using the bisBenzimide DNA Quantitation Kit (Fluorescence Assay). A standard curve was generated using calf thymus DNA standards ranging from 0 to 100 μg/mL, as shown in S7. For each sample, 200 μL of the bisBenzimide reagent was added to 50 μL of either the sample or standard in a black 96-well plate to minimize background fluorescence. The plate was incubated in the dark at room temperature for 5 minutes to allow for binding between the bisBenzimide dye and the DNA. Fluorescence was measured at an excitation wavelength of 360 nm and an emission wavelength of 460 nm using a BioTeck Synergy hybrid plate reader. The DNA concentration was calculated by comparing the fluorescence intensity to the standard curve.

The percentage of DNA reduction was calculated using the equation below:

$$\text{DNA Reduction (\%)} = \left(\frac{\text{DNA Concentration}_{\text{feed}} - \text{DNA Concentratoin}_{\text{permeate}}}{\text{DNA Concentration}_{\text{feed}}}\right) \times 100$$

All samples were analyzed in triplicate, and the results were averaged to determine the efficiency of DNA removal during the filtration process.
